# Evaluation of a multi-faceted diabetes care program including community-based peer educators in Takeo province, Cambodia, 2007-2013

**DOI:** 10.1371/journal.pone.0181582

**Published:** 2017-09-25

**Authors:** Dawn Taniguchi, James LoGerfo, Maurits van Pelt, Bessie Mielcarek, Karin Huster, Mahri Haider, Bernadette Thomas

**Affiliations:** 1 Department of Medicine, Division of General Internal Medicine, University of Washington, Seattle, Washington, United States of America; 2 Department of Global Health, University of Washington, Seattle, Washington, United States of America; 3 MoPoTsyo Patient Information Centre, Phnom Penh, Cambodia; 4 Department of Public Health, Division of Health Services, University of Washington, Seattle, Washington, United States of America; The University of Tokyo, JAPAN

## Abstract

**Introduction:**

Early detection and treatment for diabetes are essential for reducing disability and death from the disease. Finding effective screening and treatment for individuals living with diabetes in resource-limited countries is a challenge. MoPoTsyo, a Cambodian non-governmental organization, addressed this gap by utilizing a multi-pronged approach with community-based peer educators, access to laboratory procedures, local outpatient medical consultation, and a revolving drug fund. This study evaluated outcomes of MoPoTsyo’s diabetes program in Takeo Province by assessing glycemic and blood pressure outcomes for individuals diagnosed with diabetes over a 24-month follow-up period between 2007–2013.

**Methods:**

This is a retrospective cohort analysis of records without a comparison group. We calculated the mean fasting blood glucose (FBG) and blood pressure (BP) at regular intervals of follow-up. The proportion of patients reaching recommended treatment targets for FBG and BP was assessed.

**Results:**

Of the 3411 patients enrolled in the program, 2230 were included in the study. The cohort was predominantly female (68.9%) with a median age of 54 years. Median follow-up time in the program was 16 months (4.9–38.4 months). Mean FBG decreased 63.9 mg/dl in mean FBG (95% CI 58.5 to 69.3) at one year of follow-up (p<0.001). After one year, 45% (321/708) of patients achieved goal FBG < 126. Of the 41.6% (927/2230) with elevated BP at enrollment, systolic and diastolic BP levels significantly decreased (p<0.001) by 16.9 mmHg (95% CI 1.2 to 22.9) and 10 mm Hg (95% CI 0.7 to 12.9) respectively between enrollment and one year of follow-up. At one year of follow-up, 51.1%% (183/355) of these patients reached the BP goal < 140/90.

**Conclusion:**

The improved outcome indicators of diabetes care for MoPoTsyo’s Takeo program evaluation showed promise. The program demonstrated a reasonable and practical approach to delivering effective diabetes care in a rural area and may serve as a model for other low-income communities. Future prospective evaluations with more complete data are necessary for longer-term outcomes.

## Introduction

It is estimated that 387 million people are living with diabetes mellitus worldwide [1]. This number is expected to double by 2030, affecting approximately one in every ten people [2]. Left untreated, diabetes is associated with an increased risk of serious chronic complications that include vision loss, nerve damage, kidney failure, cardiovascular disease and stroke, which can all ultimately lead to early disability and premature death. Low and middle-income countries (LMIC) account for 80% of people globally living with this disease [3]. Even though early detection and treatment are essential for reducing the diabetes burden, about half of the people with diabetes remain undiagnosed [2]. Finding effective ways of improving screening, access, quality of care, and support for patients living with diabetes in LMIC is a well-known and difficult challenge [4].

Cambodia is a LMIC country in Southeast Asia with an overall diabetes prevalence of about 3% of the adults ages 25–65 years in 2010 [5]. There are few non-governmental organizations, private health clinics, or ministry of health clinics in Cambodia that implement programs to address screening, management, and treatment of diabetes [6–10] and the majority of those enrolled in a diabetes treatment program typically have poorly controlled diabetes [11].

Peer support care models may provide ways to address some of these challenges. These programs utilize peer educators who are nonprofessionals to provide a variety of functions including social and emotional support, assistance with disease management, and linkage to clinical care and community resources [12]. The main goal is to engage patients in self-management of their disease to sustain behaviors needed to manage diabetes and decrease risk of diabetes complications. Although peer support is recognized by a substantial amount of research [12–15], there is still much to learn about how to best to organize and deliver effective peer support programs. Furthermore, there are few program evaluations currently assessing the outcomes of diabetes programs [16]. This lack of programmatic monitoring and evaluation delays and impedes broader implementation of effective programs.

In 2004, MoPoTsyo Patient Information Centre (MoPoTsyo), a Cambodian non-governmental organization, was established to help address this gap in care. MoPoTsyo initially focused on improving access to education and screening through a peer educator network model, then gradually expanded to involve the management of care and treatment for individuals with diabetes in low resource settings through access to local medical outpatient consultation, laboratory testing, and revolving drug fund. A recent cross-sectional study comparing MoPoTsyo with two similar diabetes programs in Democratic Republic Congo and Philippines demonstrated MoPoTsyo to have the most favorable health outcomes, specifically in regards to glucose and blood pressure control [17]. The purpose of this study is to further describe MoPoTsyo’s diabetes program in Takeo Province, Cambodia by assessing glycemic and blood pressure (BP) outcomes over 2 years of follow-up.

## Methods and materials

### Study design

This was a retrospective cohort study of individuals with diabetes enrolled with the MoPoTsyo Program in Takeo from 2007 to 2013. The University of Washington Institutional Review Board (HSD #47688, 2014) and the Cambodian National Ethics Committee for Human Research (NECHR # 106, 2014) approved this study. Both institutional review boards provided waiver of participant consent.

### Regional description

Throughout the time of this study, Cambodia was classified as a low-income country. Takeo province is predominantly rural with a total population of 844,906 in 2008 according to the census by the Ministry of Planning. In 2009 approximately 50% of individuals were between the ages of 15 to 49, 9% between 50 and 64, and 5% older than 64 years old. The majority of employed persons were working in agriculture, forestry, and fishing. Publicly provided health care in Takeo was divided over 5 operational districts, each one with its own health authority, a referral hospital and health centers typically without physicians. During the period 2007–2013, there was not yet a role for health centers in chronic care provision.

### General program description

MoPoTsyo is a Cambodian non-governmental organization based in Phnom Penh, Cambodia. It provides care for adults with diabetes and hypertension through community-based peer educators and access to local medical outpatient consultation, laboratory testing, and a revolving drug fund. MoPoTsyo focuses on providing care for Cambodia’s most impoverished communities, including Phnom Penh’s urban slums and rural communities. It started its first rural program in Takeo Province about 100km south of Phnom Penh in 2007. That same year, MoPoTsyo set up its first peer educator network with 1 peer educator for each of the 10 health centers of Ang Roka operational district. In 2009 MoPoTsyo expanded to 4 more operational districts to complete coverage of the whole Takeo province. As of 2013, there were 56 community peer educator’s active in 56 of the 72 Health Center area (78%). During the interval 2007 through 2013, the peer educators screened reached a total of 301,049 adults in the province (about 75% of adults) identifying and registering 3,411 persons with diabetes.

#### Peer educator selection and training

MoPoTsyo’s candidate peer educators were selected from persons with diabetes based on their ability to read and write and their willingness to commit to fulfill the role. Two thirds were male and levels of education varied widely. A training manual in Khmer was created by MoPoTsyo to educate peer educators about diabetes and hypertension. The diabetes manual informed peer educators about the basic biology of diabetes, medication management, nutrition, and other lifestyle modifications. Candidate peer educators received a total of 6 weeks of training prior to testing needed to become a program peer educator. The first two weeks of training took place in Phnom Penh and included practical training by experienced urban peer educators and theoretical training by medical students hired by MoPoTsyo. The candidate peer educators then received two weeks of field training in screening for diabetes and counseling. The last two weeks allowed for time to revisit and review the knowledge and skills to prepare for a pre-exam. Once they passed the program’s pre-exam, they were offered the final exam. Those who passed the pre-exam but failed the final exam had an opportunity to practice and work as a peer educator supervised by a more experienced peer educator until they were ready to retake the final exam.

#### Peer educator activities

Peer educators screened travelled from house-to-house in each village screening for the presence of diabetes. To screen for diabetes, MoPoTsyo used urine glucose test strips. Peer educators handed out these testing strips to households and set up a follow-up appointment to check for positive results. Patients with positive urine screens were then tested using blood glucose levels to confirm the diagnosis of diabetes. MoPoTsyo used glucometers testing capillary blood for a fasting blood glucose (FBG) level greater than 126mg/dl or postprandial blood glucose (PPBG) level greater than 180 mg/dl to diagnose diabetes. Blood pressure was measured using a manual sphygmomanometer. Patients with previously diagnosed diabetes, glucose testing meeting diabetes criteria, or on anti-diabetic medication, were also offered enrollment in the program. A subset of patients who enrolled into MoPoTsyo had been previously enrolled in the Médecins Sans Frontières diabetes clinic at the provincial hospital in Takeo prior to its closure in 2009 and were under treatment at time of enrollment.

After enrollment, the peer educators facilitated patient self-management of their chronic disease to improve glycemic and BP control. In order to accomplish this, peer educators met with patients individually or in a group setting monthly for the first year in the program to provide ongoing diabetes education, support, and to check FBG levels and BP. In addition, patients were encouraged to use urine glucose strips every two weeks to self-monitor their diabetes control. MoPoTsyo encouraged peer educators to create group meetings. Given the low levels of reimbursement for their work, the large coverage area and workload, the peer educators had considerable flexibility in deciding how to spend their time with the patients. Peer educators typically organized participant groups at their homes on fixed days. Some peer educators visited every village in the health center coverage area to follow up one or more patients individually.

#### Other program services

After 2010 MoPoTsyo included access to laboratories for blood and urines tests to screen for complications of diabetes including renal function, protein in urine, and lipid levels. The laboratory results were sent to the corresponding peer educator who then explained the results to the patient. Since diabetes outpatient care was not part of the government provided care, MoPoTsyo hired a physician from Phnom Penh to travel to referral hospitals in order to provide medical consultation according to a fixed schedule, usually once or twice a month per hospital.

In order for patients to access prescribed routine medication, MoPoTsyo purchased high quality, low cost medicine and distributed them under contract to private pharmacies located next to the public hospital, for the operation of a Revolving Drug Fund. The patients paid for the full cost of medication. A detailed description of the system has been published elsewhere [18]. Patients paid a small fee for each service they used. On average, the total cost for patient services and medications cost 5.23 USD per patient per month.

### Data collection

This study evaluated data from a cohort of MoPoTsyo patients with diabetes in Takeo, Cambodia. Takeo was selected for this evaluation since it was the first rural MoPoTsyo program with the longest longitudinal data. This cohort of patients included currently or previously enrolled members of MoPoTsyo between 2007 and 2013 with diabetes alone or diabetes and hypertension.

At the time of enrollment, baseline data were gathered by peer educators and included “date of enrollment”, “age”, “gender”, “height”, “weight”, “fasting blood glucose level”, “blood pressure”, “previous diagnosis of diabetes”, and “current medications”.

In the first year in the program, patients were offered monthly peer educator follow-up visits, during which peer educators collected data on FBG levels and BP. The FBG was collected using glucometers testing capillary blood from a finger prick. Additionally, patients had FBG and/or BP data collected if they were seen by a medical doctor or referred for further lab testing. The laboratory collected venous blood to assess glucose levels. In all settings, fasting was self-reported by the patient. Altogether, follow-up data on FBG levels and BP were obtained during either peer educator, physician, or lab visits.

Data from these visits were collected in writing at the time of the patient encounter and then were entered into a central program database periodically.

### Study population

We identified all individuals with diabetes with or without high BP enrolled from February 1, 2007 to July 1, 2013. Patients were included if they were over 18 years of age and had an initial FBG, at least one follow-up FBG, and an initial BP check. Patients under the age of 18 were excluded from the analysis.

### Descriptive analysis

We used descriptive statistics (proportion, median, 25^th^ and 75^th^ quartiles) to describe characteristics of the cohort at enrollment. Student T-tests and chi-squared tests were used as appropriate to assess if there were a significant difference in age, sex, FBG, SBP, and DBP between the included and excluded groups. Significance was defined as an alpha level of 0.05.

### Glucose analysis

We had four main objectives for the FBG analysis. First, we assessed the cohort’s longitudinal trend of mean FBG levels over 2 years of follow-up. In order to assess this change in FBG levels over time of program enrollment, we trended the mean FBG levels from enrollment until designated follow-up intervals of 6, 12, 18, and 24 months. A follow-up interval was defined as the approximate amount of months an individual was enrolled in the program at the time the data was collected. For uniformity we recalibrated the follow-up time period. Therefore, the term “6 months” indicates a range of follow-up between 0 and 6 months, “12 months” ranged between 6 and 12 months in program, “18 months” ranged between 12 and 18 months, and so forth. Second, we observed the change in individual FBG levels after one year of enrollment in the program. One year of follow-up was felt to be an adequate amount of time to see the initial effects of the program’s interventions on FBG. We used the paired t-test to compare this mean difference in a patient’s paired FBG value at baseline and one year of follow-up. Third, we looked at the proportion of patients within the program’s FBG goal at the previously designated follow-up intervals. We assessed this by calculating the proportion of the cohort that met the recommended target for FBG level at regular intervals after enrollment in the program. We used two FBG goals for this target analysis. The first was the program’s goal FBG of less than or equal to 126 mg/dl. The second was a FBG goal of less than 150 mg/dl since this has been shown to approximate HbA1c < 7 [19]. Last, we assessed the difference in mean FBG at enrollment, 6 months, and 12 months of follow-up between patients reported to be on diabetes medication and those not on medications at time of enrollment using paired t-test.

### Blood pressure analysis

In our analysis of BP management we first assessed the cohort’s longitudinal trend of mean SBP and DBP levels over 2 years of follow-up. We then decided to narrow our interest group to individuals with elevated systolic (SBP ≥ 140 mm Hg) or diastolic (DBP ≥ 90 mm Hg) BP at the time of enrollment. By focusing on patients with hypertension at baseline, our overall goal was to assess the program’s ability to improve BP control in this higher risk group. In order to do this, we identified individuals with elevated systolic or diastolic BP at baseline and omitted patients with BPs that were within goal (<140/90 mm Hg) [20]. For the group of patients with elevated BP, we trended the mean SBP and DBP at each follow-up interval to assess the program’s long-term effects on BP over time. The paired t-test was used to compare the mean difference between paired BP values at baseline and 12 months of follow-up. Similar to the FBG analysis, one year of follow-up was felt to be an adequate amount of time to assess the program’s initial effects on BP. We evaluated the proportion of this cohort that met the recommended target for BP at regular intervals after enrollment. A BP target of less than 140/90 mm Hg was used.

Data analysis was performed using STATA software (College Station, TX), version 11 and Microsoft Excel 2011 (Redmond, WA).

## Results

### Patient characteristics

**Fig 1:**
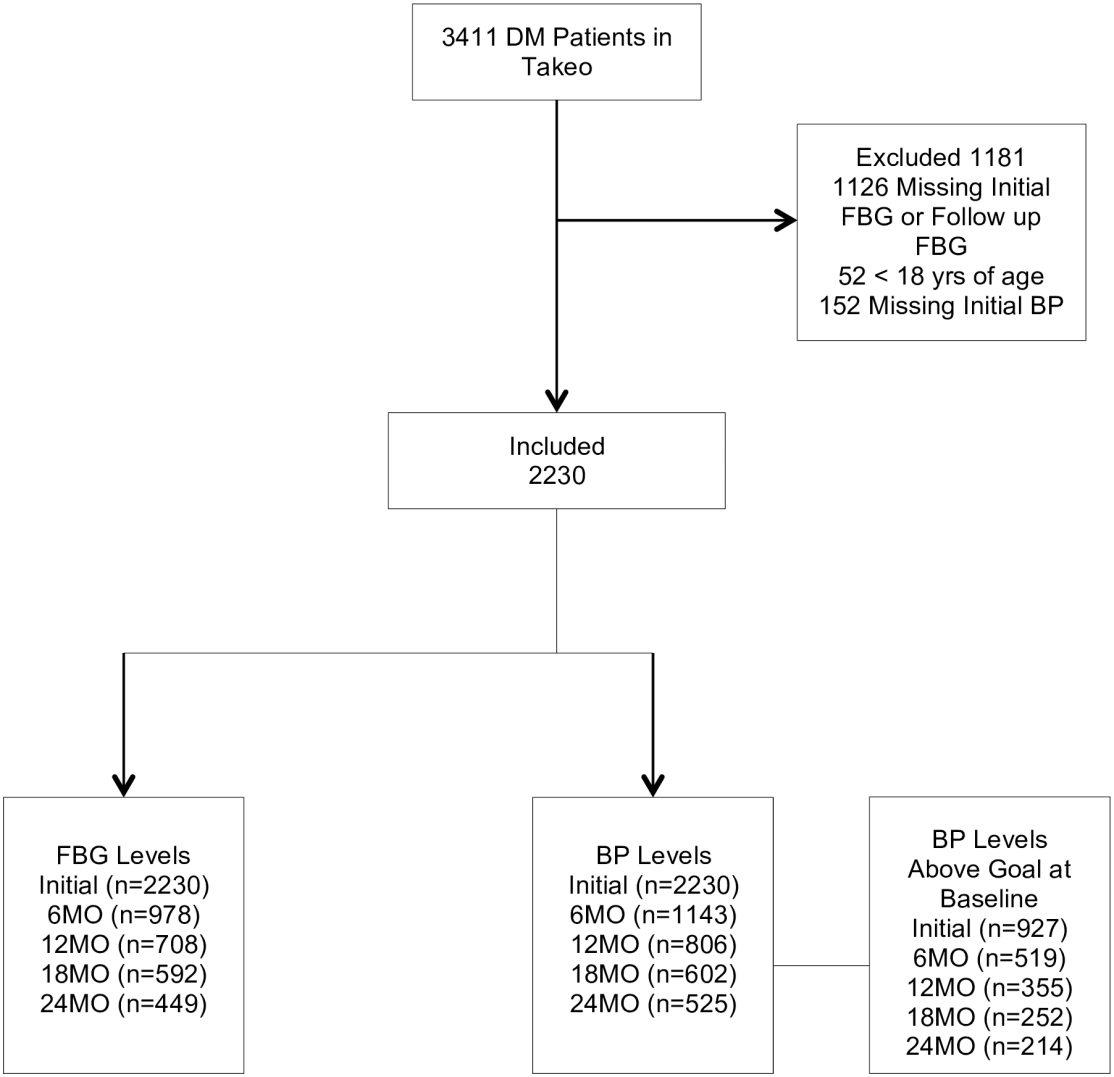
Flow chart of included and excluded patients.

A total of 3411 patients were enrolled in the Takeo MoPoTsyo program between February 1, 2007 and July 1, 2013 with either newly diagnosed or known diabetes. Of these, 2230 (66.7%) were included in the data analysis (Fig 1). The demographic characteristics of the 2230 individuals included in the study are presented in Table 1. Patients were predominantly female (68.9%) with a median age of 54.5 years. The largest proportion of individuals was between the ages of 50 to 59 (34%). Only about 10% of the cohort was between the ages of 18 to 39. The median follow-up time in the program was 16 months (4.9–38.4 months). At time of enrollment, 22.4% (218/975) of patients reported being on medications for DM and 19.6% (96/489) reported being on high blood pressure medications. At the end of the study period, 90.1% were still active in the program, 27 (1.2%) had died, and 193 (8.7%) were lost to follow-up.

**Table 1:** Characteristic of the DM patients at initial visits.

Characteristic	
Total no. of patients with DM	2230
Sex—Women, n(%)	1538 (68.9%)
Age in years, median (IQR)	54.5 (46.3, 61.9)
Age Group in Years, n (%)	
18–39	233 (10.4%)
40–49	555 (24.9%)
50–59	758 (34%)
60–69	509 (22.8%)
≥ 70	175 (7.8%)
FBG (mg/dl), median (IQR)	195 (152, 255)
FBG < 126 mg/dl, n (%)	223 (10%)
Systolic BP (mm Hg), median (IQR)	130 (118, 146)
Diastolic BP (mm Hg), median (IQR)	82 (74, 91)
BMI (kg/m2), median (IQR) (n = 2229)	23.1 (20.6, 25.5), Missing 1*
BMI < 23 kg/m2, n (%)	1091 (48.9%)
Treatment at Admission	
Diabetes Treatment, n (%) (n = 975)	218 (22.4%), Missing 1355*
High Blood Pressure Treatment, n (%) (n = 489)	96 (19.6%), Missing 1741*
Follow-up Duration in months, median (IQR)	16.2 (4.9, 38.5)
Active, n (%)	2010 (90.1%)
Died, n (%)	27 (1%)
Lost, n (%)	193 (8.7%)

*missingness

### Inclusion/Exclusion

A total of 1181 people were excluded from the analysis due to age less than 18 (52/3411), missing initial, or follow-up FBG levels (1126/3411) or missing initial BP measurements (152/3411). The individuals excluded from the analysis had a significantly lower (p<0.05) number of women (61.6% versus 68.9%) and a higher initial FBG (226.6 mg/dl versus 215.9 mg/dl) (Table 2). There was no significant difference in age, initial SBP or DBP between groups.

**Table 2:** Characteristics of included and excluded DM patients.

Characteristic	ALL (n = 3411)	Included (n = 2230)	Excluded (n = 1181)
Sex—Women, n(%)*	2266 (66.4%)	1538 (68.9%)	728 (61.6%)
Age in years, mean (IQR)	54.3 (11.9)	54.2 (11.2)	54.5 (13.1)
FBG Level (mg/dl), mean*	219.4 (90.8)	215.9 (89.2)	226.6 (93.7)
SBP (mm Hg), mean	133.7 (22.9)	133.5 (22.5)	134.1 (23.9)
DBP (mm Hg), mean	83.3 (13.1)	83.1 (12.8)	83.6 (13.9)

Results indicate mean values unless otherwise indicated.

Parentheses indicate standard deviation unless otherwise indicated.

*Indicates statistically significant association between Included and Excluded Patients (p < 0.05)

### Glucose control

**Fig 2:**
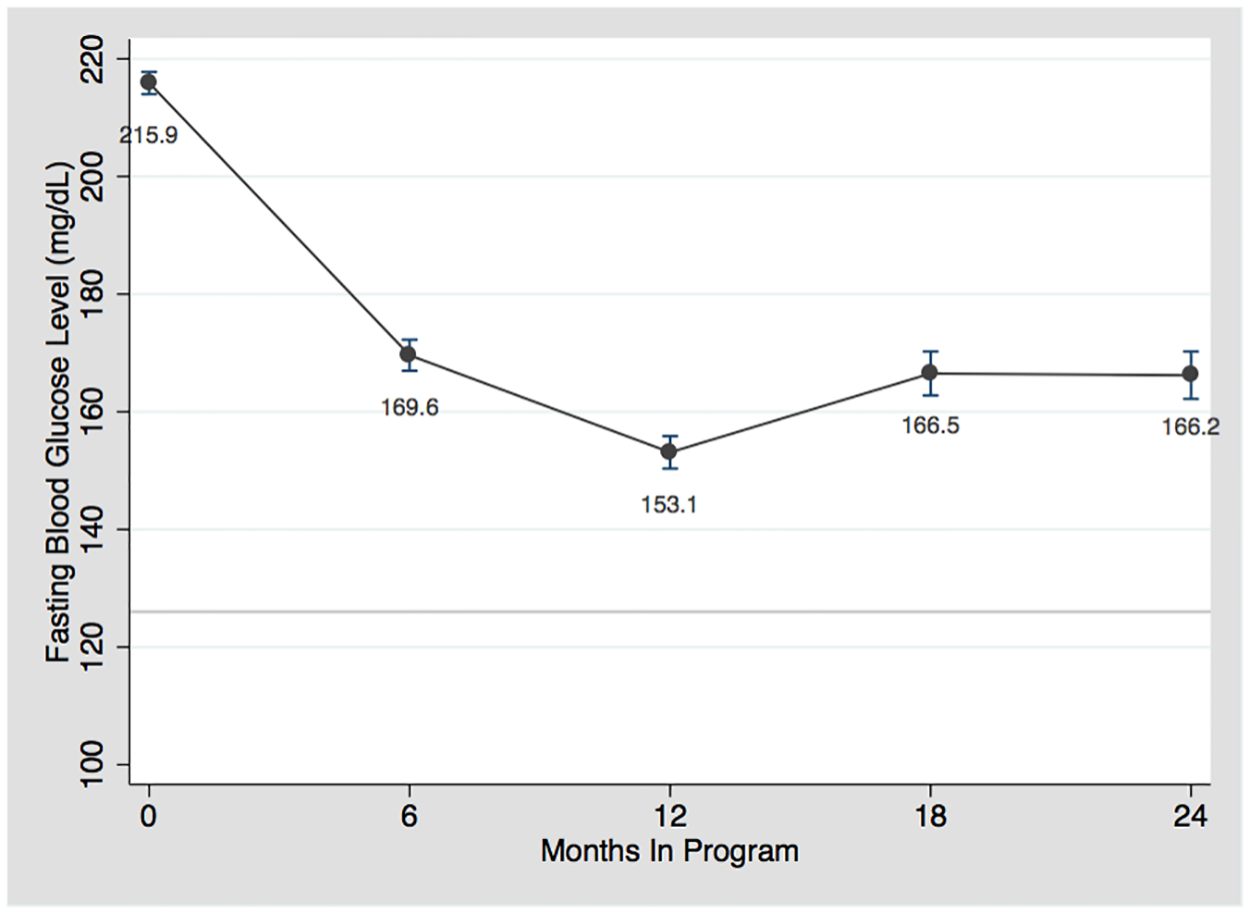
Mean fasting blood glucose level over time.

Fig 2 illustrates the trend in the mean values for FBG over time in the program. There was a significant decrease (p<0.001) of 63.9 mg/dl in mean FBG (95% confidence interval (CI) 58.5 to 69.3) at one year of follow-up when compared to enrollment FBG (Table 3). The initial decrease seen in FBG at 6 and 12-month follow-up points was sustained over the 2 years of follow-up and approached goal FBG of less than 126 mg/dl.

**Table 3:** Mean difference between paired observations at baseline and month 12 in patients with baseline and follow-up values.

Variable	Mean Difference	95% CI	p-value
FBG (n = 1325)	63.9 mg/dl	(58.5 to 69.3)	<0.001
SBP (n = 355)	16.9 mm Hg	(1.2 to 22.9)	<0.001
DBP (n = 355)	10 mm Hg	(0.7 to 12.9)	<0.001

For SBP, only patients with elevated SBP at baseline (SBP ≥ 140 mmHg) were included in the analysis. For DBP, only patients with elevated DBP at baseline (DBP ≥ 90 mmHg) were included in the analysis.

**Fig 3:**
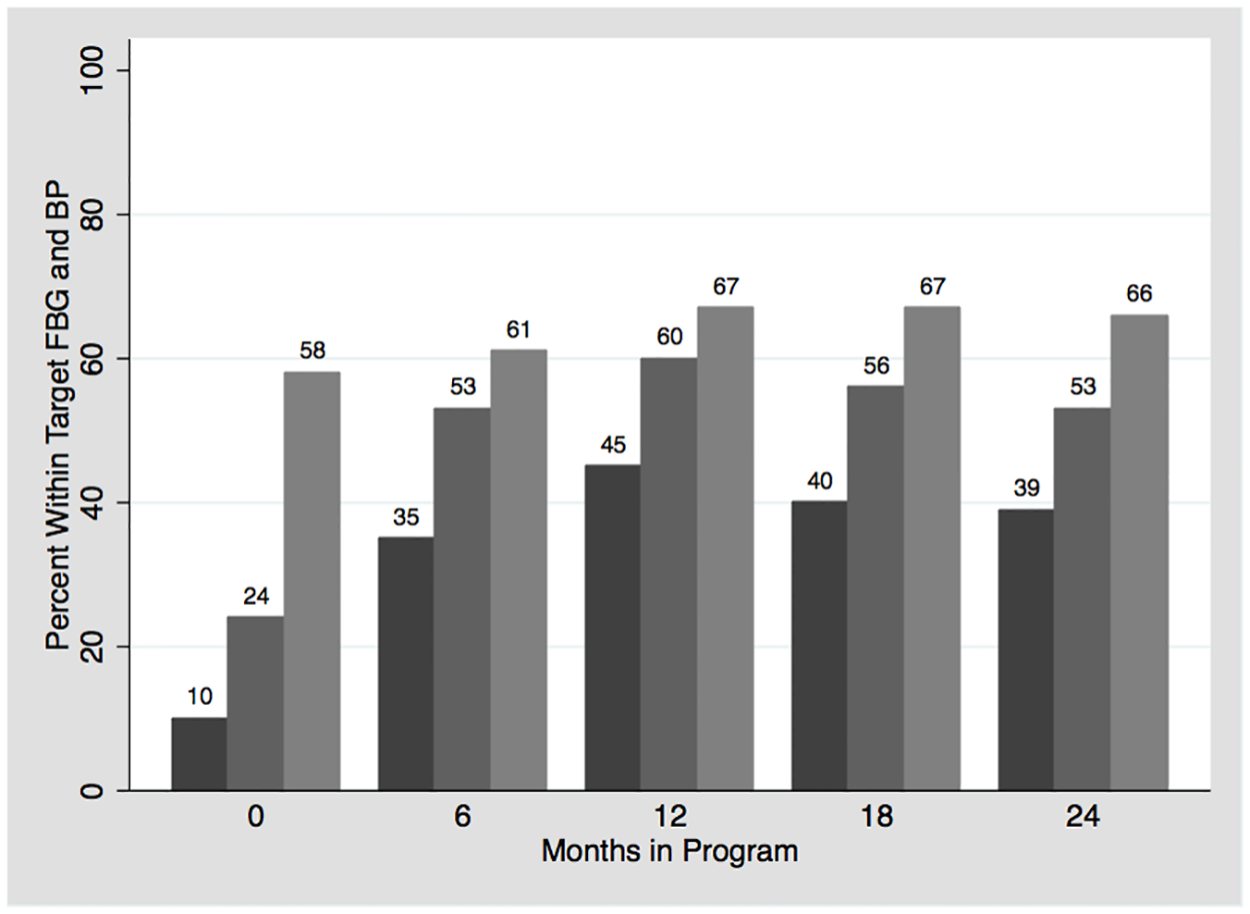
Proportion of patients reaching recommended target.

The proportion of patients reaching the recommended targets for glucose is shown in Fig 3 and Table 4. At enrollment, only 10% of patients had a FBG less than 126 mg/dl. After one year, 45% (321/708) of patients achieved this goal. When assessing goal FBG less than 150 mg/dl, 60% and 53% were within goal at one year and two years of follow-up, respectively.

**Table 4:** Proportion of patients within FBG goal at follow-up intervals; using FBG goal of < 126 and < 150.

Month in Program	Proportion of patients within FBG Goal (FBG <126)	Proportion of patients within FBG Goal (FBG < 150)
0 (n = 2230)	10%	24.1%
6 (n = 978)	35.1%	52.6%
12 (n = 708)	45.3%	60.1%
18 (n = 592)	40.4%	56.4%
24 (n = 499)	39.2%	53.4%

The mean FBG for patient on diabetes medications at enrollment was initially 216 mg/dl and decreased to 163 mg/dl and 159 mg/dl at 6 and 12-month follow-up, respectively. The calculated mean FBG for patients not on diabetes medication at enrollment was initially 241 mg/dl and decreased to 171 mg/dl and 148 mg/dl at 6 and 12-month, respectively. By 6 months of follow-up, the two groups’ mean FBG were not significantly different (p>0.05).

Each follow-up interval for glucose was a range. For the “6 month” interval, the mean follow-up time was 2.8 months (SD 1.9). The mean follow-up time for 12 months, 18 months, and 24 months was 9.4 (SD 1.6), 15.3 (SD 1.7), and 21.3 (SD 1.7) months, respectively.

### Blood pressure control

Similar to the FBG analysis, each follow-up interval for blood pressure was a range. For the “6 month” interval, the mean follow-up time was 3.3 months (SD 1.9). The mean follow-up time for 12 months, 18 months, and 24 months was 9.7 (SD 1.7), 15.7 (SD 1.7), and 21.5 (SD 1.7) months, respectively.

**Fig 4:**
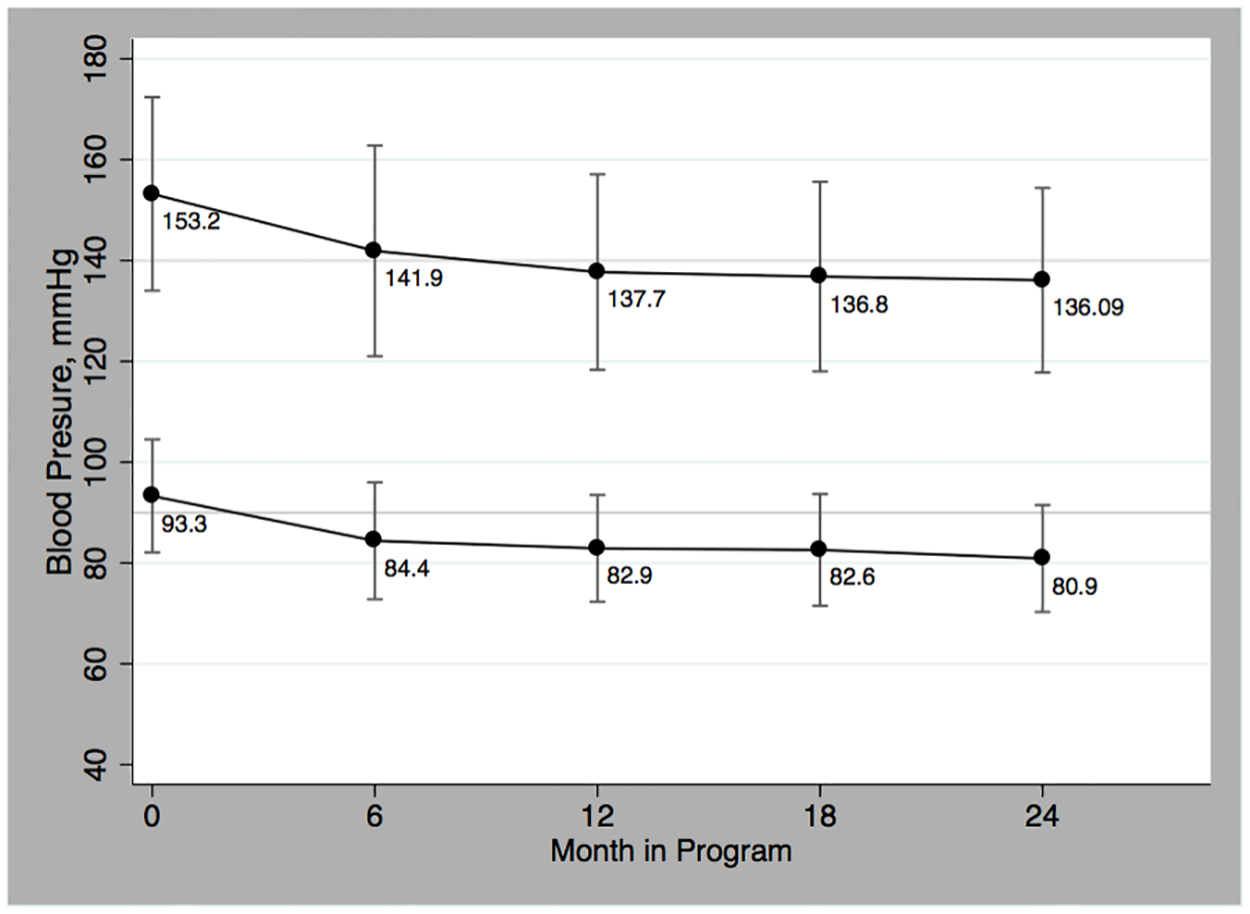
Mean blood pressure over time.

In Fig 4, the decreasing trend in mean SBP and DBP is shown for patients with elevated SBP and/or DBP at enrollment (927/2230). In this group the systolic and diastolic levels significantly decreased (p<0.001) by 16.9 mmHg (95% CI 1.2 to 22.9) and 10 mm Hg (95% CI 0.7 to 12.9) respectively between enrollment and one year of follow-up (Table 3). As seen in the FBG analysis, the initial mean SBP and DBP decrease seen at 6 and 12-month follow-up was maintained over the 2 years and approached goal BP of less than 140/90 mmHg.

The proportion of patients reaching the recommended targets for BP is shown in Fig 3 and Table 5. When assessing BP control, 58.4% (1303/2230) of all patients had a BP less than 140/90 mm Hg at enrollment. Of the 41.6% (927/2230) with elevated BP at enrollment, 51.5% (183/355) of them reach the BP goal at one year of follow-up. When accounting for all patients, 65.7% (345/525) had target BP at 2 years of follow-up.

**Table 5:** Proportion of patients within BP goal at follow-up intervals; using BP of less than 140/90.

Month in Program	Proportions of all patients within BP goal (<140/90 mm Hg)	*Proportions of patients within BP goal (BP<140/90 mm Hg)
0	58.4% (n = 2230)	n/a
6	61.2% (n = 1143)	44.1% (n = 519)
12	67.2% (n = 806)	51.5% (n = 355)
18	67.1% (n = 602)	53.6% (n = 252)
24	65.7% (n = 525)	52.8% (n = 214)

*This analysis only includes patients with elevated BP at baseline (BP ≥140/90 mm Hg)

## Discussion

The outcome indicators of diabetes care for MoPoTsyo’s Takeo program evaluation are promising. Both FBG levels and BP measurements showed an improvement within six months of enrollment and maintained these improved levels of control over 2 years of follow-up. After one year in the program, the proportion of patients reaching the target FBG level tripled and over half of the patients with uncontrolled BP at enrollment were within goal by this one-year mark. This illustrates a potential approach to delivering effective quality diabetes care to a large number of patients in rural parts of LMICs by reducing existing barriers to care thereby increasing access to diagnosis, treatment, laboratory testing, and providing disease education and support for patients.

The heterogeneity of the existing literature makes it difficult to construct meaningful comparisons to other programs [16]. Studies in the literature are typically much smaller in size with shorter follow up periods [21, 22]. It is also challenging to directly compare MoPoTsyo to programs utilizing community health care workers because MoPoTsyo includes additional patient support by offering improved access to medications, laboratory services, and physician consultation. In our study the decrease of FBG from 216 mg/dl to 163 mg/dl represents a decrease of 32%. A study of more intensively trained community health workers following a structured protocol in India revealed a change from 214 mg/dl to 180mg/dl or 25% decrease in newly diagnosed patients [23]. In that study, there was a strong focus on lifestyle change and included access to medication management. A follow up study with a less intense intervention showed a decrease of 14.9 mg/dl from a baseline of 166mg/dl in persons with diabetes [24].

The majority of other published diabetes program evaluations in LMICs are assessments of hospital and clinic based programs typically having more immediate accessibility to physicians, laboratory testing, and medications. In these studies the proportion of patients reaching target glucose levels ranged from 24% to 39% suggesting inadequate control for the majority of patients in treatment [16]. For example, a hospital-based program in Cambodia estimated 24% of their patients were within HbA1c goal of less than 7% [9]. In the neighboring country of Thailand, a middle income country, patients managed in two clinic-based programs reached HbA1c of less than 7% in 24% and 32% of their patients [25]. A government run diabetes clinic in Sri Lanka revealed less than a third (30%) of patients had a HbA1c of less than 7.5% [26]. In Malaysia, an assessment of several hospitals, diabetes clinics, and specialty clinics revealed less than one in four patients had reached the recommended target HbA1c of less than 7% [27].

There are several possible explanations for MoPoTsyo’s encouraging outcomes. MoPoTsyo focused on pro-active community screening for early detection of disease. This potentially allowed for patients to receive care earlier in the disease process. This may be one reason a recent study revealed MoPoTsyo to have favorable patient characteristics including younger age and lower BMI when compared to two other programs [17]. MoPoTsyo offered access to medical care and medications at a total cost of 5.23 USD per patient per month. This potentially allowed for increased adherence and compliance in treatment recommendations. MoPoTsyo also had a strong emphasis on patient self-management by immersing patients during their first year in frequent diabetes education sessions and community building for social support. The slight rise in FBG levels at 18 and 24 months could be attributed to the fewer peer educators follow-up visits after the first year and increasing cost of services borne by the patient. Overall, the effectiveness of MoPoTsyo’s comprehensive diabetes care is reflected in their improved control of both patients on diabetes medications and not on treatment at time of enrollment. Finally, the use of peer educators who live in the community and also have to overcome barriers to behavior change and health system access may have played a significant role in the level of effectiveness despite variable levels of structured teaching.

This study has several limitations. First, data were drawn from the program’s central database that was not originally designed for research purposes. Potential missteps in gathering data and data entry errors could have occurred. Second, about a third of the individuals with diabetes enrolled in the Takeo program were excluded from the study due to missing data, which could have introduced selection bias. Third, there was a lack of a comparison group therefore it is not known if individuals with diabetes in other programs or receiving care from health centers alone did equally as good over this time period. Fourth, at the time of the study the program did not keep data on which components of the program enrollees participated in therefore it is difficult to know which interventions were most instrumental to improvements in outcomes. Fifth, there is a lack of physician standardization of care in the MoPoTsyo program. Medications for diabetes and hypertension are prescribed at the discretion of the treating physician from a restrictive list of generic medications available in the program’s revolving drug fund. Last, in this study we used FBG as an indicator of diabetes control due to the available data. Fasting was determined by patient report. HbA1c is a better predictor of management of control in many parts of the world [19]. Using FBG may have underestimated the proportion of patients within goal since a HbA1c of 7% translates to approximately a FBG level of 150 mg/dl [19]. Also, patients may have falsely reported fasting. Despite these limitations, this study’s strengths include its large cohort size, prospective data collection, and assessment of community-based program implementation.

As of 2016, the Cambodian Ministry of Health has incorporated the model in the Cambodian National strategic plan for non-communicable diseases.

## Conclusion

The improved outcome indicators of diabetes care for MoPoTsyo’s Takeo program evaluation showed promise. The program demonstrated a reasonable and practical approach to delivering effective diabetes care in a rural area and may serve as a model for other low-income communities. Future prospective evaluations with more complete data are necessary for longer-term outcomes.

## Supporting information

S1 File This is the fasting blood glucose dataset. This is the data used for the fasting blood glucose analysis.
(DTA)

S2 File This is the blood pressure dataset. This is the data used for the blood pressure analysis.
(DTA)

## Abbreviations

BP: blood pressure
CI: confidence interval
DBP: diastolic blood pressure
FBG: fasting blood glucose
LMIC: low and middle-income countries
MoPoTsyo: MoPoTsyo Patient Information Centre
PPBG: postprandial blood glucose
SBP: systolic blood pressure
USD: United States dollars
