# Correction: Evaluation of a multi-faceted diabetes care program including community-based peer educators in Takeo province, Cambodia, 2007-2013

**DOI:** 10.1371/journal.pone.0196192

**Published:** 2018-04-17

**Authors:** Dawn Taniguchi, James LoGerfo, Maurits van Pelt, Bessie Mielcarek, Karin Huster, Mahri Haider, Bernadette Thomas

Fig 3 is incomplete. The authors have provided a corrected version here.

**Fig 3:**
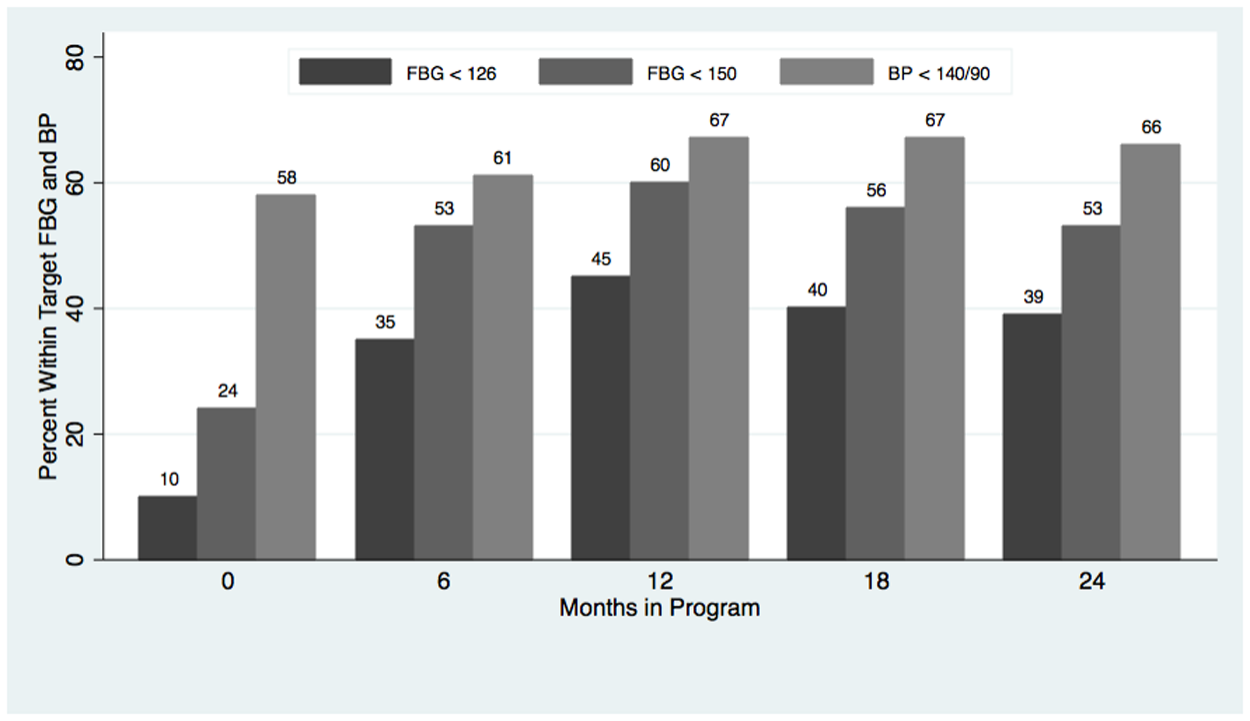
Proportion of patients reaching recommended target.
